# A systematic review of necrotising fasciitis in children from its first description in 1930 to 2018

**DOI:** 10.1186/s12879-019-3941-3

**Published:** 2019-04-11

**Authors:** Arne Schroder̈, Aurelié Gerin, Gregory B. Firth, Kelly S. Hoffmann, Andrew Grieve, Christina Oetzmann von Sochaczewski

**Affiliations:** 1 Klinik für Anästhesiologie und Intensivmedizin, Marienkrankenhaus Bergisch-Gladbach, Dr.-Robert-Koch-Straße 18, Bergisch-Gladbach, D-51465 Germany; 2 Department of Paediatrics, Chris Hani Baragwanath Academic Hospital, Faculty of Health Sciences, University of the Witwatersrand, 26 Chris Hani Road, Johannesburg, ZA-1860 South Africa; 3 Department of Orthopaedic Surgery, Chris Hani Baragwanath Academic Hospital, Faculty of Health Sciences, University of the Witwatersrand, 26 Chris Hani Road, Johannesburg, ZA-1860 South Africa; 4 Department of Paediatric Surgery, Universitair Medisch Centrum Groningen, Hanzeplein 1, Groningen, NL-9713 The Netherlands; 5 Department of Paediatric Surgery, Chris Hani Baragwanath Academic Hospital, Faculty of Health Sciences, University of the Witwatersrand, 26 Chris Hani Road, Johannesburg, ZA-1860 South Africa; 6 Klinik und Poliklinik für Kinderchirurgie, Universitätsmedizin Mainz, Langenbeckstraße 1, Mainz, D-55131 Germany

**Keywords:** Necrotising fasciitis, Children, Systematic review, Incidence rate, Case fatality rate, Predisposing factors, Symptoms

## Abstract

**Background:**

Necrotising fasciitis is a rapidly progressing soft-tissue infection with a low incidence that carries a relevant risk of morbidity and mortality. Although necrotising fasciitis is often fatal in adults, its case fatality rate seems to be lower in children. A highly variable clinical presentation makes the diagnosis challenging, which often results in misdiagnosis and time-delay to therapy.

**Methods:**

We conducted a protocol-based systematic review to identify specific features of necrotising fasciitis in children aged one month to 17 years. We searched ’PubMed’, ’Web of Science’ and ’SCOPUS’ for relevant literature. Primary outcomes were incidence and case fatality rates in population-based studies, and skin symptoms on presentation. We also assessed signs of systemic illness, causative organisms, predisposing factors, and reconstructive procedures as secondary outcomes.

**Results:**

We included five studies reporting incidence and case fatality rates, two case-control studies, and 298 cases from 195 reports. Incidence rates varied between 0.022 and 0.843 per 100,000 children per year with a case-fatality rate ranging from 0% to 14.3%. The most frequent skin symptoms were erythema (58.7%; 175/298) and swelling (48%; 143/298), whereas all other symptoms occurred in less than 50% of cases. The majority of cases had fever (76.7%; 188/245), but other signs of systemic illness were present in less than half of the cohort. Group-A streptococci accounted for 44.8% (132/298) followed by Gram-negative rods in 29.8% (88/295), while polymicrobial infections occurred in 17.3% (51/295). Extremities were affected in 45.6% (136/298), of which 73.5% (100/136) occurred in the lower extremities. Skin grafts were necessary in 51.6% (84/162) of the pooled cases, while flaps were seldom used (10.5%; 17/162). The vast majority of included reports originate from developed countries.

**Conclusions:**

Clinical suspicion remains the key to diagnose necrotising fasciitis. A combination of swelling, pain, erythema, and a systemic inflammatory response syndrome might indicate necrotising fasciitis. Incidence and case-fatality rates in children are much smaller than in adults, although there seems to be a relevant risk of morbidity indicated by the high percentage of skin grafts. Systematic multi-institutional research efforts are necessary to improve early diagnosis on necrotising fasciits.

**Electronic supplementary material:**

The online version of this article (10.1186/s12879-019-3941-3) contains supplementary material, which is available to authorized users.

## Background

Necrotising fasciitis is a rapidly progressing soft-tissue infection, which has historically been linked to penetrating trauma in war times [1]. Paediatric textbooks did not mention necrotising fasciitis before 1973 [2, 3] despite the first case of necrotising fasciitis in a child [4] being reported just six years after the initial description in adults [5]. Selective literature reviews dealing with necrotising fasciitis in childhood usually deduce their recommendations from small case series or reports on adults [6–9]. In them, considerable research effort has been made to analyze necrotising fasciitis on a population based level [10], for specific patient groups at risk for necrotising fasciitis [11], and to facilitate early diagnosis [12–14].

In contrast, the knowledge on paediatric necrotising fasciitis is scarce: One database article identified 334 children with necrotising soft-tissue infections, but focused on treatment, outcome, and a multivariate analysis of independent risk factors for fatal outcomes [15]. The two largest studies reporting on skin signs, risk factors and outcomes include 39 retrospectively assessed [16] and 32 prospectively included cases [17]. The 39 retrospective cases were collected within 30 years [16], whereas the prospective study was conducted within four years, but included 20 neonates [17]. The difference in research on necrotising fasciitis in adults and children may further be emphasised by studies on laboratory parameters that may aid in diagnosis of necrotising fasciitis: While 20 children were investigated in a case-control study [18], a meta-analysis of adult patients included 846 cases from 16 studies [14]. Recently, a systematic review of necrotising fasciitis in children has been published [19], which is hampered by several shortcomings: Limited to articles published in English language after 2010, lack of clearly defined inclusion and exclusion criteria, inclusion of neonates, and cases likely to be Fournier’s gangrene due to genital involvement. Therefore, we aimed to identify specific features of necrotising fasciitis in childhood that may aid in early diagnosis and treatment initiation of this devastating disease by means of a systematic review. Furthermore, we aimed to gather information on causative organisms and the necessity of reconstructive procedures following an episode of necrotising fasciitis in children.

## Methods

### Guidelines and protocol for the systematic review

We developed a Preferred Reporting Items for Systematic Reviews and Meta-Analyses - Protocols [20] compliant protocol (Additional file 1) for the systematic review, and closely followed the Preferred Reporting Items for Systematic Reviews and Meta-Analyses guidelines [21] during the systematic review.

### Literature search strategy

The literature search strategy with its adaptations to the three databases ’PubMed’, ’Web of Science’ and ’SCOPUS’ is laid out in detail in the appendix of the protocol (Additional file 1). A sensitivity-oriented approach combining text elements and Medical Subject Headings was used in all three databases. Literature search was extended towards snowballing the reference lists of all included studies and relevant reviews. We conducted the literature search on the 9^th^ of January 2016 and updated it at the 4^th^ of December 2018.

### Types of included studies

Preliminary searches failed to identify prospective studies on signs on presentation. We therefore opted to include retrospective case series and case reports to collect information on these aspects, because information from reports with higher quality were not available. Only population-based data were considered eligible to determine incidence and case-fatality rates.

### Inclusion criteria

Inclusion criteria for our systematic review were: Patient age between one month and 17 years. Studies have reported symptoms on presentation separate for each patient or for the whole group if all cases were within the age limit. Studies should indicate whether risk factors were present. Studies have reported on case fatalities. Studies reporting on incidence and case fatality rates on a population-based level must include data within the same age limits as stated above, but do not have to report on signs at presentation or risk factors.

### Exclusion criteria

Exclusion criteria for our systematic review were: Studies were narrative reviews. Studies include patient data outside the specified age group that cannot be removed from the reported results. Studies include data on neonates or Fournier’s grangrene that cannot be separated from the paediatric data. Studies were reported in languages that could not be adequately translated using Google translator into a language that one of the authors can speak fluently (English, German, French, Dutch/Afrikaans, and Spanish).

### Primary and secondary outcomes

Our primary outcomes were: Determine incidence and case-fatality rates of necrotising fasciitis in children from population-based reports and assess skin symptoms on presentation. Our secondary outcomes were: Age-specific case fatality rates, risk factors for necrotising fasciitis, signs of systemic illness due to necrotising fasciitis, microbes causative for necrotising fasciitis and reconstructive procedures following necrotising fasciitis.

### Literature selection and data extraction

Two researchers independently assessed the search results and extracted data from the included reports as described in the protocol (Additional file 1). Following de-duplication, titles were independently screened for eligibility followed by reading the abstracts as second and the full-text as a third step. Each step was checked for consistency by another researcher. Differences between the two independent researchers were settled by consensus. If consensus could not be reached, the assessment of a third researcher was decisive. We used a Data extraction sheet (Additional file 2) for the documentation of the results.

### Definitions for data acquisition

We defined all skin symptoms which were not explicitly mentioned in a report as absent. This definition was also used for signs of systemic illness not reported. Signs of systemic illness and reconstructive procedures were only included if at least one item was reported in the study, otherwise the respective cells were not included in the analysis. Definitions for the systemic inflammatory response syndrome relied on the international paediatric sepsis consensus conference [22].

### Risk factors in the pooled cases

We grouped the underlying conditions or preceding events of the included cases into five distinct risk groups: Varicella, surgery, immunocompromise, trauma, and minor trauma (e.g. an insect bite, a bruise from a fall etc.) and contrasted them with the cases in which necrotising fasciitis occurred without predisposing factors.

### Protocol deviations

The study by *Mulla* reports cases of necrotising fasciitis in children caused by group-A streptococci in Florida between August 1996 and August 2000, but did not provide incidence data [23]. We extrapolated incidence data by using census data of Florida in 2000, which counted 3,646,340 persons below 18 years of age [24]. The population data used in the Finnish incidence study [25] had an age limit of 15 years. The neonatal case in the study by *Eneli & Davies* [26] has been excluded and the incidence data were recalculated using population data provided within the report. The report by *Gjessing Jensen & Christensen* [27] was not translated using Google translator as stated in the protocol, because the corresponding author supplied us with an author translation.

## Results

### Article selection

**Fig. 1:**
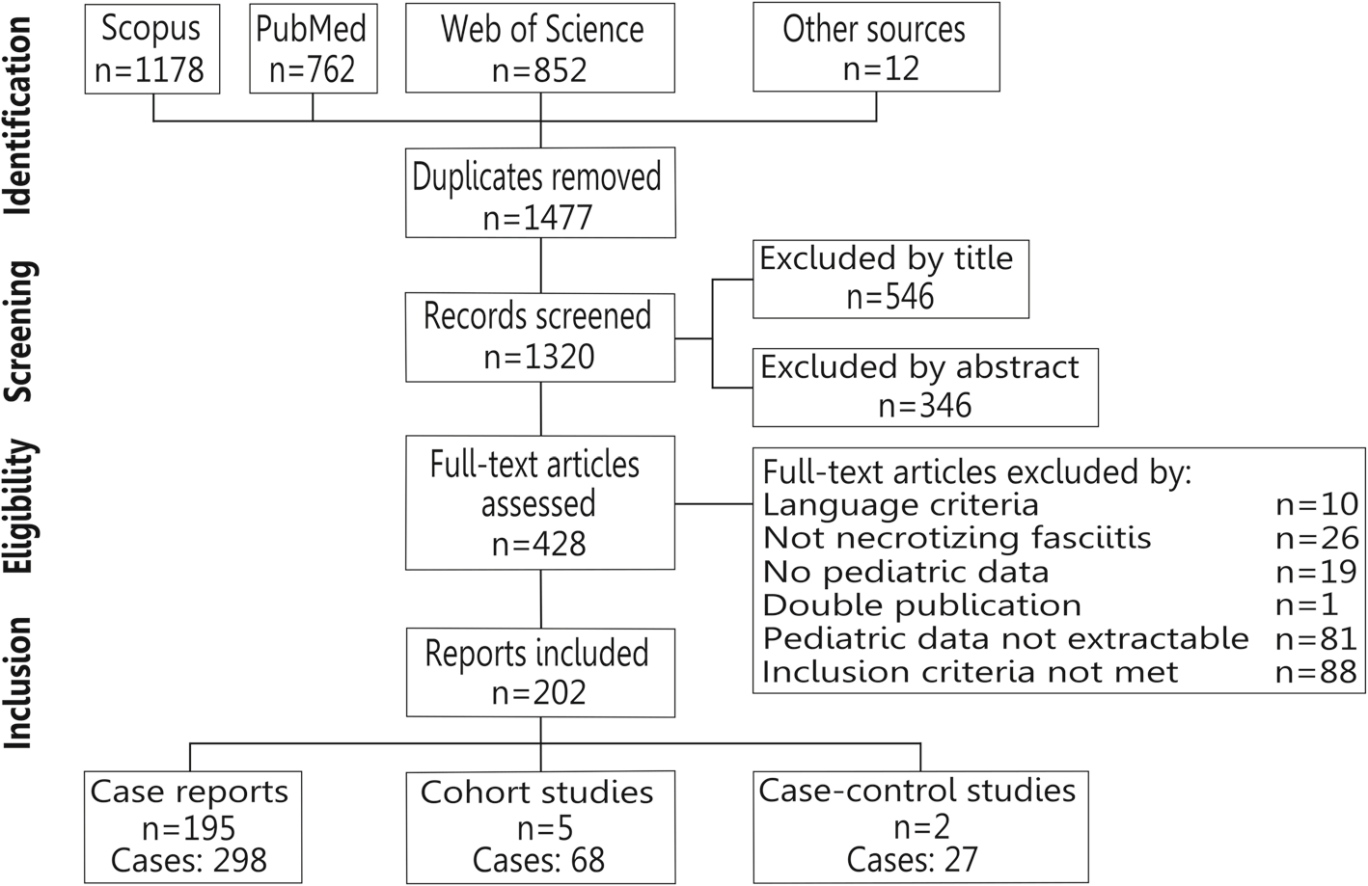
Preferred reporting items for systematic reviews and meta-analyses flow diagram. Literature search and selection of studies for the systematic review

We identified five studies that reported population-based on incidence and case-fatality rates in 68 cases [23, 25, 26, 28, 29], two case-control studies with 27 cases [30, 31], and another 298 cases from 195 case series and case reports [2–4, 8, 27, 32–221] (Fig. 1).

### Incidence rate

Two studies from Canada were prospective: One monitored the whole country [26], whereas the other was limited to Ontario [28]. Another study collected retrospective data for Florida [23] and another relied on the database of a hospital chain in Utah, which claimed to cover 70-85% of all hospital admissions of children in this state [29]. The last report was based on the data of Finnish university hospitals and the childhood population living in their referral area [25]. Only one study [26] reports incidence data for cases of necrotising fasciitis for both group-A streptococci and non-group-A streptococci, whereas the remaining three studies were limited to necrotising fasciitis caused by group-A streptococci [23, 28, 29]. The incidence rate of necrotising fasciitis varied from 0.843 cases per 100,000 children per year due to group-A streptococci in the Finnish Oulu University Hospital area [25] to 0.022 cases per 100,000 children per year caused by group-A streptococci [23]. Incidence rates of 0.212 cases occurred due to group-A streptococci and 0.0729 cases were caused by all other organisms [26] 0.01 [29] and 0.08 per 100,000 children per year [28] were in between.

### Case fatality rate

The case fatality rate differed between 14.3% (1/7) [29], 10% (1/10) [28], and 0% in two reports [(0/3) [23] and (0/13) [25]], but was 2.85% (1/35) in the only study that included cases caused by other germs than group-A streptococci [26].

### Properties of the identified case-control studies

The first identified case-control study had a mixed design of retrospectively (5/19) and prospectively (14/19) included cases. It aimed to describe an association between necrotising fasciitis following primary varicella infection and a preceding treatment with ibuprofen [30]. Twenty-nine controls were prospectively identified and had non-necrotising skin infections following primary varicella infection [30]. Therefore, study parameters were collected with the intent to compare baseline variables between two groups [30]. The second case-control study aimed to identify specific features of necrotising fasciitis compared to non-necrotising soft tissue infections [31]. It included cases within 16 years [31], whereas the first study had a duration of 19 months [30].

### Age, sex and geographic distribution

The included cases had a similar mean age compared to the case-control studies (Table 1). Distribution of age groups within the pooled cases was similar except for a slight over-representation of school children and a corresponding under-representation of adolescents and infants. Males were predominantly affected in the varicella and ibuprofen case-control study with 74% (14/19) [30], and within the pooled cases (57.4%, 171/298), but not in the second case-control study (3/8 males) [31]. North America accounted for 39.9% (119/298), Asia for 31.9% (95/298), and Europe for 21.1% (63/298) of the included cases. In contrast, South America contributed ten (3.3%), Africa nine (3%), and Oceania only two reports.

**Table 1:** Age, risk factors, skin symptoms, and signs of systemic illness in case-control studies and pooled cases

Item	Zerr et al. [30]	Hsieh et al. [31]	Pooled cases [2–4, 8, 27, 32–221]
Age [years] (range)	4.6 (0.5-9.6)	5 (2-13)	5.7 (0.1-17)
Varicella [%] (Number)	100 (19/19)	50 (4/8)	25.9 (77/297)
No risk factor [%] (Number)	0	12.5 (1/8)	22.9 (68/297)
Minor trauma [%] (Number)	0	0	12.5 (37/297)
Immunocompromise [%] (Number)	0	0	11.8 (35/297)
Surgery [%] (Number)	0	12.5 (1/8)	9.4 (28/297)
Trauma [%] (Number)	0	25 (2/8)	8.1 (24/297)
Skin infection [%] (Number)	0	0	6.1 (18/297)
Intramuscular injection [%] (Number)	0	0	1.7 (5/297)
Neuropathy [%] (Number)	0	0	1.4 (4/297)
Diabetes [%] (Number)	0	0	0.4 (1/297)
Erythema [%] (Number)	94.7 (18/19)	87.5 (7/8)	58.7 (175/298)
Swelling [%] (Number)	100 (19/19)	87.5 (7/8)	48 (143/298)
Pain [%] (Number)	100 (19/19)	87.5 (7/8)	33.6 (100/298)
Splinting [%] (Number)	66.7 (12/18)	37.5 (3/8)	3 (9/298)
Tenderness [%] (Number)	0	37.5 (3/8)	25.2 (75/298)
Discolouration [%] (Number)	0	0	32.6 (97/298)
Necrosis [%] (Number)	0	0	32.2 (96/298)
Oedema [%] (Number)	0	0	26.5 (79/298)
Induration [%] (Number)	0	0	14.4 (43/298)
Warmth [%] (Number)	0	0	10.7 (32/298)
Bullae [%] (Number)	0	0	9.1 (27/298)
Discharge [%] (Number)	0	0	8.4 (25/298)
Ecchymosis [%] (Number)	0	0	8.4 (25/298)
Blister [%] (Number)	0	0	6 (18/298)
Crepitus [%] (Number)	0	0	3.7 (11/298)
Fever [%] (Number)	100 (19/19)	Not reported	76.7 (188/245)
Tachycardia [%] (Number)	0	Not reported	40.7 (94/231)
Tachypnea [%] (Number)	0	Not reported	27.7 (64/231)
Hypotension [%] (Number)	26.3 (5/19)	Not reported	29.9 (69/231)
Leukocytosis [%] (Number)	Not reported	Not reported	49.5 (106/214)
Bandemia [%] (Number)	Not reported	Not reported	22.9 (49/214)
Leukopenia [%] (Number)	Not reported	Not reported	17.3 (29/214)
Hypothermia [%] (Number)	0	Not reported	0.8 (2/245)
Bradycardia [%] (Number)	0	Not reported	0.4 (1/231)
Systemic inflammatory response syndrome [%] (Number)	Not reported	Not reported	65.1 (175/269)

### Items assessed in the systematic review

The results can be found in Tables 1 and 2. An itemisation for risk factors revealed that there were no obvious differences between them.

**Table 2:** Isolated germs, involved body regions, fatalities, and reconstructive procedures in case-control studies and pooled cases

Item	Zerr et al. [30]	Hsieh et al. [31]	Pooled cases[2–4, 8, 27, 32–221]
Group-A streptococci [%] (Number)	84.2 (16/19)	87.5 (7/8)	44.8 (132/295)
*Staphylococcus aureus* [%] (Number)	5.3 (1/19)	12.5 (1/8)	18.6 (55/295)
Gramnegative rods combined [%] (Number)	0	0	29.8 (88/295)
*Pseudomonas aeruginosa* [%] (Number)	0	0	10.2 (30/295)
*Escherichia coli* [%] (Number)	0	0	7.8 (23/295)
*Serratia marcescens* [%] (Number)	0	0	1.7 (5/295)
*Klebsiella* species [%] (Number)	0	0	1.7 (5/295)
Other gramnegative rods [%] (Number)	0	0	8.5 (25/295)
Anaerobe microbes [%] (Number)	0	0	7.1 (21/295)
Other streptococci [%] (Number)	0	0	6.8 (20/295)
Fungi [%] (Number)	0	0	3.4 (10/295)
Other staphylococci [%] (Number)	0	0	3.1 (9/295)
*Enterococcus* species [%] (Number)	0	0	2.7 (8/295)
Polymicrobial infection [%] (Number)	5.3 (1/19)	0	17.3 (51/295)
Extremities [%] (Number)	63.2 (12/19)	Not reported	45.6 (136/298)
Lower extremity [%] (Number)	Not reported	Not reported	33.9 (100/298)
Upper extremity [%] (Number)	Not reported	Not reported	12.1 (36/298)
Trunk [%] (Number)	21.1 (4/19)	Not reported	32.9 (98/298)
Head [%] (Number)	15.8 (3/19)	Not reported	20.8 (62/298)
Retroperitoneum [%] (Number)	0	Not reported	0.7 (2/298)
Second body region involved [%] (Number)	0	Not reported	16.8 (50/298)
Lower extremity [%] (Number)	0	Not reported	11.1 (33/298)
Trunk [%] (Number)	0	Not reported	3.7 (11/298)
Upper extremity [%] (Number)	0	Not reported	2 (6/298)
More than two body regions involved [%] (Number)	0	Not reported	2.7 (8/298)
Fatalities [%] (Number)	0	0	10.4 (31/295)
Primary closure [%] (Number)	Not reported	Not reported	17.3 (28/162)
Secondary closure [%] (Number)	Not reported	Not reported	20.4 (33/162)
Skin graft [%] (Number)	Not reported	Not reported	51.6 (84/162)
Skin flap [%] (Number)	Not reported	Not reported	10.5 (17/162)

## Discussion

We aimed to identify features specific to necrotising fasciitis in children by a systematic review. Whereas necrotising fasciitis had been subject to intensive research efforts in adults, knowledge on necrotising fasciitis in children is scarce. We identified four studies reporting population based incidences and case-fatality rates, of which two were prospectively conducted. Moreover, three of them were limited to cases of necrotising fasciitis caused by group-A streptococci and had a narrow geographic focus: Either a Canadian province [28], states within the United States of America [23, 29] or the referral area of Finnish university hospitals [25]. Only one report assessed necrotising fasciitis on a nationwide level [26] and included cases caused by other organisms than group-A streptococci. This might represent an accurate estimation of the burden of disease for an industrialised country. Necrotising fasciitis may be more common in developing countries as indicated by a monocentric Nigerian prospective observational study: It included 32 cases of necrotising fasciitis in childhood within 4 years [17]. Although 20 cases were neonates - leaving 12 children - the report [17] still hints at higher incidences in low-income countries. Moreover, all of these children presented with tissue necrosis [17] indicating an advanced disease [9, 222]. Similarly, ecchymosis and necrosis were found in 72% of cases in the other large case series [16]. In contrast to these late-appearing skin symptoms, pain, erythema, swelling, and - to a lesser extent - splinting were predominant skin symptoms in both case-control studies [30, 31]. The analysis of skin symptoms within the pooled cases did not identify highly frequent lesions: Only erythema had been noted in more than a half of the included cases and swelling in almost a half of the affected cases despite an advanced stage of disease - evidenced by either ecchymosis or necrosis - in 40.6% of the cases. This result may be explained by recall bias: The lack of clinical information that has been present in the patient, but was not documented in the patient’s file and thus not included in the published report [223]. Consequently, the more subtle skin symptoms might not be documented in light of the more dramatic changes such as necrosis or discolouration. Recall bias is also likely to have affected the signs of systemic illness within the pooled cases. Signs of systemic illness had similar frequencies among the pooled cases with the exception of fever in 76.6%. This percentage was smaller than the 100% reported in both case-control studies, and the 92% in the largest report on paediatric necrotising fasciitis [16]. Frank et al. [6] suggested that necrotising fasciitis would often go hand in hand with normal white cell counts combined with pronounced bandemia, whereas others associated necrotising fasciitis specifically with increased white cell counts [9]. In both case-control studies, white cell counts did not differ from those in patients diagnosed with cellulitis [30, 31]. Within the pooled cases, 49.5% had leukocytosis, 17.3% leukopenia, and 22.9% bandemia, of which the majority were found conjointly with leukocytosis. However, leukocyte counts are not part of the paediatric laboratory risk indicator for necrotising fasciitis, developed to differentiate cellulitis from necrotising fasciitis: Only C-reactive protein and sodium levels below 135mmol/L were found to be of relevance [18]. An abnormal leukocyte count or temperature are required to diagnose systemic inflammatory response syndrome, which occured in 65.1% of the pooled cases. A recent case-control study has shown that fever, tachycardia, and tachypnea might be used to differentiate necrotising fasciitis from abscesses or cellulitis [224]. Thus, a systemic inflammatory response syndrome conjointly with the combination of the most frequent skin symptoms from the case-control studies - swelling, pain, erythema, and probably splinting - might be predictive for necrotising fasciitis. Due to the limitations of the data included in the systematic review, this symptom combination needs to be evaluated for its predictive value before recommendations can be made. Predominant involvement of extremities followed by lesions on trunk and head was a common picture within all included studies. It also is in line with other reports [16] and adult data [11, 225, 226]. This is different concerning polymicrobial necrotising fasciitis: Previously, polymicrobial infection was commonly reported in paediatric necrotising fasciitis in developed [227] as well as developing countries [16, 17]. Among the pooled cases, in contrast, necrotising fasciitis was usually monomicrobial, which has previously been attributed to necrotising fasciitis following primary varicella infection [30, 31, 222]. Whether the pooled cases provide an accurate estimation of the distribution of mono- and polymicrobial infections needs to be assessed at a larger scale. In particular, Gram-negative rods isolated from wounds may depict a changing spectrum of necrotising fasciitis with a transition of risk factors from primary varicella towards immunocompromised or operated patients. Introduction of varicella vaccination resulted in reduction of the case load of necrotising fasciitis caused by group-A streptococci [228, 229]. Again, these results have to be validated by large scale studies. The necessary information seem to be available in certain databases as the negative association of both *Streptococcus* spp. and *Staphylococcus* spp. with case fatalities [15] could not have been calculated without knowledge of isolated germs. Case fatality rates have been [225] and still are high in adults [10, 226], but lower or absent in children [16–18, 222, 227, 230]. Higher case fatality rates of 14.3% [29] and 10% [28] have likely been influenced by small sample sizes as the case fatality rate was only 2.85% in the only cohort with more than ten patients [26]. Case fatality rate was 10.4% within the pooled cases and thus higher than in the aforementioned studies. Similarly, the number of cases that required a skin graft was 51.6%, which largely exceeds the previously reported values of skin grafting [17, 230]. There has been considerable variation within the literature: From skin grafts being exceptional [222] to institutions where skin grafting is the regular treatment modality for skin defects following necrotising fasciitis [227]. Probably, necessity for skin grafts was determined by extent of debridement and may thus have influenced the number of skin grafts. Different thresholds for using skin grafts could also play a role. Besides the already mentioned recall bias, several other limitations need to be taken into account for data from case series and case reports. Usually, case reports and series have an exorbitantly high success rate. Either due to preferential reporting of successful results [231] or an over-representation of specialised centres [232], whereas terrible results are scarce and those in between almost non-existent. The extent of this bias is however unclear as an assessment of case series included in Health Technology Assessments of the National Institute of Clinical Excellence of the United Kingdom found no differences in reported outcomes compared to randomised controlled clinical trials on the same subject [233]. Nevertheless, the results from the pooled cases have to be interpreted cautiously and thus require validation by studies of higher quality. Despite the relevance of necrotising fasciitis and its potential grave consequences for the future life of children, these studies are missing. Likely due to the rarity of necrotising fasciitis for the individual institutions, which could be overcome by multiinstitutional collaboration.

## Conclusions

A high index of suspicion is necessary to diagnose necrotising fasciitis. A combination of swelling, pain, erythema, and a systemic inflammatory response syndrome might be indicative of early stages of necrotising fasciitis. Incidence and case-fatality rates of necrotising fasciitis in childhood are much smaller than in adults. Nevertheless, necrotising fasciitis seems to carry a relevant risk of morbidity exemplified skin grafting in more than a half of the pooled cases. A systematic multiinstitutional research effort is necessary to gain meaningful results from future studies to further elucidate necrotising fasciitis in childhood.

## Additional files

Additional file 1 Preferred reporting items for systematic reviews and meta-analyses-protocol compliant systematic review protocol. Protocol for the systematic review. (PDF 259 kb)

Additional file 2 Data extraction sheet. Sheet used for data extraction and documentation. (PDF 8 kb)

Additional file 3 Dataset for the pooled cases. Complete database of all cases extracted from the literature and their coding for the respective items. (XLSX 91 kb)
