# Correction to: A systematic review of necrotising fasciitis in children from its first description in 1930 to 2018

**DOI:** 10.1186/s12879-019-4003-6

**Published:** 2019-05-27

**Authors:** Arne Schröder, Aurélie Gerin, Gregory B. Firth, Kelly S. Hoffmann, Andrew Grieve, Christina Oetzmann von Sochaczewski

**Affiliations:** 1 Klinik für Anästhesiologie und Intensivmedizin, Marienkrankenhaus Bergisch-Gladbach, Dr.-Robert-Koch-Straße 18, 51465 Bergisch-Gladbach, Germany; 2 Department of Paediatrics, Chris Hani Baragwanath Academic Hospital, Faculty of Health Sciences, University of the Witwatersrand, 26 Chris Hani Road, Johannesburg, ZA-1860 South Africa; 3 Department of Orthopaedic Surgery, Chris Hani Baragwanath Academic Hospital, Faculty of Health Sciences, University of the Witwatersrand, 26 Chris Hani Road, Johannesburg, ZA-1860 South Africa; 4 Department of Paediatric Surgery, Universitair Medisch Centrum Groningen, Hanzeplein 1, Groningen, NL-9713 The Netherlands; 5 Department of Paediatric Surgery, Chris Hani Baragwanath Academic Hospital, Faculty of Health Sciences, University of the Witwatersrand, 26 Chris Hani Road, Johannesburg, ZA-1860 South Africa; 6 Klinik und Poliklinik für Kinderchirurgie, Universitätsmedizin Mainz, Langenbeckstraße 1, 55131 Mainz, Germany

**Correction to: BMC Infect Dis**

**https://doi.org/10.1186/s12879-019-3941-3**

After publication of the original article [1], we were notified that two of the author names were incorrectly displayed in the pdf version of the paper, while one other name was incorrectly tagged in the XML version. Below you can find the changes:

- Arne Schrö should be replaced with Arne Schröder
- Aurelié Gerin should be replaced with Aurélie Gerin
- Oetzmann von Sochaczewski should be tagged as the full family name for Christina Oetzmann von Sochaczewski

The original article has been corrected.
