# Supplementary material for: A systematic review of necrotising fasciitis in children from its first description in 1930 to 2018
Source: BMC Infect Dis. 2019 Apr 11;19:317. doi: 10.1186/s12879-019-3941-3 (PMC6458701; doi:10.1186/s12879-019-3941-3)
Supplement: Supplementary file 1 — Preferred reporting items for systematic reviews and meta-analyses-protocol compliant systematic review protocol. Protocol for the systematic review. (PDF 259 kb) [file 12879_2019_3941_MOESM1_ESM.pdf]

Necrotizing fasciitis in the pediatric population: A protocol for a systematic review

Background

Necrotizing fasciitis is a rare disease, in adults as well as in children with a 0.08 incidence per 100.000 children per year in the United States, but often fatal [1]. First described in ancient times, it had been present throughout all wars fought by humankind, which resulted in its association to penetrating injuries and Gram-positive streptococcus pyogenes [2]. In adults, the case fatality rate varies considerably between different studies that reported values between 6 and 76% with an average of 34% [3]. Interestingly, the amount of fatal cases seems to be much lower in the pediatric population [4]. Diagnosis remains a challenge due to the highly variable presentation with a high range of possible combinations of cutaneous symptoms and possible differential diagnoses. The Laboratory Risk Indicator for Necrotizing Fasciitis (LRINEC) has been developed to address the diagnostic challenge with its high positive predictive value of 92% [5]. However, its results could not be reproduced in different patient cohorts despite several optimizations by including different laboratory values and clinical signs [6].Others relied on clinical factors and their combination to achieve the diagnosis of necrotizing fasciitis [7]. However, none of these tests has been validated in a pediatric population. As they all include clinical signs, the prevalence within the target population influences the results of the tests. Reports about pediatric cases of necrotizing fasciitis are usually case reports and small, retrospective case series. So, in contrast to the various large cohorts of necrotizing fasciitis cases in adults, there is no guiding value on the presence of symptoms in a larger pediatric cohort. More uncertainty is added as consistent risk factors could not be identified between the various small reports on the pediatric population [4,8].

So far, a systematic review that specifically deals with the risk factors, symptoms and mortality in the pediatric age group has not been conducted. A systematic review of the published literature will yield synthesized data that can be used to compare the results of future studies against a reference value.

Methods

This protocol has been written according to the preferred reporting items for systematic reviews and meta-analyses protocols (PRISMA-P) statement [9].

Types of studies: A preliminary search failed to identify systematic reviews and prospective studies in children with necrotizing fasciitis. Therefore, we will include all retrospective studies including case series and case reports that report at least one case of necrotizing fasciitis in the pediatric age group. Should we identify any other suitable studies based on the criteria laid out below will they also be included if they meet the inclusion criteria. A limitation solely on case reports and case series is not intended.

Participants: All participants above the age of 1 month to the age of 17 years will be eligible. Neonates will be excluded, because necrotizing fasciitis in the neonatal period is considered a different entity of the disease [10]. Fournier's gangrene will be excluded due to this reason [11], too, including cases involving genitals or perineum due to the considerable overlap between the forms. Both excluded forms have been reviewed elsewhere [10,12,13]. Varicella gangrenosa will not be included if necrotic involvement of fascia is absent. Studies that report on both children and adults are eligible, if data was reported separately for the pediatric age group.

Inclusion criteria:

- Participant age between 1 month and 17 years.
- Studies must have reported symptoms on presentation separate for each participant or for the whole group if all cases are within the age limit stated above.
- Studies should indicate whether risk factors were present.
- Studies must have reported information on case fatality.
- Studies reporting on incidence and case fatality rate must include data within the same age limits as stated above that can be extracted, but do not have to report on presentation and risk factors.

Exclusion criteria:

- Studies were narrative reviews.

- Study results include patient data outside the specified age group that cannot be removed from the reported results.
- Includes data on neonates or Fournier's gangrene that cannot be separated from the pediatric data.
- Studies were reported in languages that could not be adequately translated with the use of google translator for available documents to extract the data into one of the languages that the authors are fluent in (English, German, French, Afrikaans/Dutch and Spanish).

Interventions: Not applicable.

Outcome measures: Primary outcome will be the prevalence of skin symptoms, associated risk factors and mortality. Secondary outcomes will be age-specific case-fatality rate, other physical findings indicating systemic illness and involved microbes.

Search strategy: We will conduct an electronic database search without restrictions concerning language or dates in MEDLINE, SCOPUS and Web of Science. A combination of text words and Medical Subject Headings (MeSH) will be used. The search strategy will be adapted to every database and is provided in detail in the Appendix. We preferred not to exclude neonates by the use of textwords or MeSH as it may exclude suitable studies when they present cases of neonatal necrotizing fasciitis and necrotizing fasciitis in other pediatric age groups. Hence, the more sensitivity oriented approach was chosen and studies dealing only with the neonatal form of necrotizing fasciitis will be excluded in the screening process. The reference lists of included studies and relevant reviews will be searched for additional studies.

Processing of search results: The search results will be imported into JabRef 3.1 (http://www.jabref.org/), a reference manager that allows deduplication of the search results.

Data collection

Selection of studies: De-duplicated results will be screened by the title whether they are eligible, excluded studies will be marked and checked by a second researcher for consistency. Reading the abstracts will be the second step to identify suitable studies. The results of abstract-reading will be checked for consistency by a second researcher.

The assessment of full-texts will be independently carried out by two researchers and checked for consistency, too. Differences between the researchers on each step will be settled by consensus, if consensus could not be reached, the assessment of a third researcher will be decisive. The results will be presented in a PRISMA flowchart.

Data extraction: Data will be extracted from the text, tables, figures and if applicable supplemental information onto a standardized data extraction form. In cases of unclear data or uncertain eligibility of a data subset differences will be solved by consensus or if it could not be reached by the decision of a third researcher. Extracted data will consist of:

- Study characteristics: period, design, objective, sample size and founding source.
- Study population: country and setting.
- Patient demographics: age(mean or median), gender, socioeconomic status.
- Skin signs of necrotizing fasciitis and their prevalence.
- Other signs of systemic illness in necrotizing fasciitis.
- Risk factors for the development of necrotizing fasciitis.
- Germs that had been obtained from infected tissue.
- Fatal cases within the study population.

Quality assessment: All included studies will be critically and independently assessed for their quality and potential flaws by two authors. However, our preliminary search yielded only case reports and case series, which are of low quality according to the GRADE guidelines [14]. Retrospective case reports and case series in general are affected by selection bias, especially if the studies are not consecutive. The second concern is information bias that result through an unblinded, possibly unstructured data collection and by the lack of information in retrospective chart review. Moreover, these studies suffer from publication bias as the interesting or complicated cases are more likely to get published.

Data synthesis

We will calculate the frequency of reported skin symptoms, physical findings and risk factors as well as outcome measures in the form of the frequency of deficits, especially concerning amputation and need for corrective surgery for wound coverage. A subgroup analysis for age may be added if the included data is suitable in a way that enough

individual cases are reported that can form the basis of a subgroup analysis according to age and geographic region of the conducted study. A possible subgrouping for age would be a differentiation between infants, toddlers, school children and adolescents. The low quality of included studies renders a quantitative analysis that exceeds the presentation of frequencies within the reported cases unsuitable. Incidence and mortality will preferably be reported from studies examining the population within larger geographic areas. Incidence will be reported as cumulative incidence and incidence rates separately for each included study as well as the case fatality rates. A comparison of interventions and treatment modalities is explicitly not intended.

Data reporting

We will report the data in a narrative review and might include a presentation of the frequencies of symptoms and the case fatality rate within the subgroups in a separate figure. We will explicitly highlight that the case fatality rates derived from the pooled, reported cases will are most likely not representative for the disease as the larger observational studies for a specified population within a specified geographic region are of a much higher quality and thus more reliable. The reporting of results will adhere to the PRISMA statement.

References:

[1] Laupland KB, Davies HD, Low DE, et al. Invasive group A streptococcal disease in children and association with varicella-zoster virus infection. Pediatrics. 2000;105:e60.

[2] Lamb LE, Sriskandan S, Tan LK. Bromine, bear-claw scratch fasciotomies, and the Eagle effect: management of group A streptococcal necrotising fasciitis and its association with trauma. Lancet Infect Dis. 2015;15:109-121.

[3] McHenry CR, Piotrowski JJ, Petrinic D, et al. Determinants of mortality for necrotizing soft-tissue infections. Ann Surg. 1995;221:558-563.

[4] Eneli I, Davies HD. Epidemiology and outcome of necrotizing fasciitis in children: an active surveillance study of the Canadian Paediatric Surveillance Program. J Pediatr. 2007;151:79-84.

[5] Wong CH, Khin LW, Heng KS, et al. The LRINEC (Laboratory Risk Indicator for Necrotizing Fasciitis) score: a tool for distinguishing necrotizing fasciitis from other soft tissue infections. Crit Care Med. 2004;32:1535-1541.

[6] Borschitz T, Schlicht S, Siegel E, et al. Improvement of a Clinical Score for Necrotizing Fasciitis: 'Pain Out of Proportion' and High CRP Levels Aid the Diagnosis. PLoS One. 2013;10:e0132775.

[7] Alayed KA, Tan C, Daneman N. Red Flags for Necrotizing Fasciitis: A Case Control Study. Int J Infect Dis. 2015;36:15-20.

[8] Fustes-Morales A, Gutierrez-Castrellon P, Duran-Mckinster C, et al. Necrotizing fasciitis: report of 39 pediatric cases. Arch Dermatol. 2002;138:893-899.

[9] Moher D, Shamseer L, Clarke M, et al. Preferred Reporting Items for Systematic Review and Meta-Analysis Protocols (PRISMA-P) 2015 statement. Syst Rev. 2015;4:1.

[10] Hsieh WS, Yang PH, Chao HC, et al. Neonatal necrotizing fasciitis: a report of three cases and review of the literature. Pediatrics. 1999;103:e53.

[11] de Roos WK, van Lanschot JJ, Bruining HA. Fournier's gangrene: the need for early recognition and radical surgical débridement. Neth J Surg. 1991;

[12] Nazir Z. Necrotizing fasciitis in neonates. Pediatr Surg Int. 2005;21:641-644.

[13] Adams JR Jr., Mata JA, Venable DD, et al. Fournier's gangrene in children. Urology. 1990;35:439-441.

[14] Balshem H, Helfand M, Schünemann HJ, et al. GRADE guidelines: 3. Rating the quality of evidence. J Clin Epidemiol. 2011;64:401-406.

Appendix

MEDLINE via PubMed

1 necrotizing fasciitis[ALL]

2 necrotizing soft tissue infect*[ALL]

3 fasciitis necrotisans[ALL]

4 necrotizing fasciitis[MH]

5 necrotizing fasciitis[TW] OR necrotising fasciitis[TW]

6 necrotizing soft tissue infect*[TW] OR necrotising soft tissue infect*[TW]

7 #1 OR #2 OR #3 OR #4 OR #5 OR #6

8 child[MH]

9 child, preschool[MH]

10 infant[MH]

11 child*[TW]

12 infant*[TW]

13 pediat*[TW] OR paediat*[TW]

14 toddler*[TW]

15 youth*[TW]

16 young people*[TW]

17 juvenile*[TW]

18 adolescent*[TW]

19 preschool*[TW]

20 #8 OR #9 OR #10 OR #11 OR #12 OR #13 OR #14 OR #15 OR #16 OR #17 OR #18 OR #19

21 #7 AND #20

Web of Science via Thomson Reuters

1 TS=("Necroti$ing fasciitis" OR "necroti$ing soft tissue infect*" OR "fasciitis necrotisans" OR (fasciitis near/5 necro*))

2 TS=(child* OR infant* OR p$diat* OR toddler* OR youth* OR juvenile* OR "young people*" OR adolescent* OR preschool*)

3 #1 AND #2

SCOPUS via Elsevier

1 TITLE-ABS-KEY({necroti?ing fasciitis} OR {necrotic?ing soft tissue infect*} OR {fasciitis necrotisans}) OR TITLE-ABS-KEY(necro* W/5 fasciitis)

2 TITLE-ABS-KEY(child* OR infant* OR pediat* OR paediat* OR toddler* OR youth* OR juvenile* OR {young people*} OR adolescent* OR preschool*)

3 #1 AND #2
