# Supplementary material for: A systematic review of necrotising fasciitis in children from its first description in 1930 to 2018
Source: BMC Infect Dis. 2019 Apr 11;19:317. doi: 10.1186/s12879-019-3941-3 (PMC6458701; doi:10.1186/s12879-019-3941-3)
Supplement: Supplementary file 2 — Data extraction sheet. Sheet used for data extraction and documentation. (PDF 8 kb) [file 12879_2019_3941_MOESM2_ESM.pdf]

Data extraction sheet for the systematic review on necrotizing fasciitis in childhood

<table>
<tr><td>Journal</td><td>Year</td><td>Volume</td><td>Pages</td><td>First Author</td><td>Study type</td><td>Duration</td></tr>
<tr><td>Institution</td><td>Geographic region</td><td>NecFasc</td><td>Cases</td><td>Pediatric</td><td>Age</td><td>Sex</td><td>Race</td></tr>
<tr><td>Inclusion criteria: (Present)</td><td>Age group</td><td>Symptoms</td><td>Systemic illness</td><td>Risk factors</td><td>Case fatality</td></tr>
<tr><td>Exclusion criteria: (Present)</td><td>Only adult data</td><td>Pediatric data not extractable</td><td>Language requirement met</td></tr>
<tr><td>Incidence of NecFasc</td><td>Definition of population at risk</td><td>Case Fatality rate</td><td>Individual cases?</td></tr>
<tr><td>Skin symptoms reported (with frequency)</td><td>Signs of systemic illness (with frequency)</td></tr>
<tr><td>Microbes reported (with frequency)</td><td>Risk factors reported (with frequency)</td></tr>
<tr><td>Body region involved (with frequency)</td><td>Outcome/Sequelae (with frequency)</td></tr>
<tr><td>Quality assessment</td><td>Laboratory data</td></tr>
<tr><td>Case number in statistical analysis</td><td>Other</td></tr>
</table>
